# PFK2/FBPase-2 is a potential target for metabolic engineering in the filamentous fungus *Myceliophthora thermophila*

**DOI:** 10.3389/fmicb.2022.1056694

**Published:** 2022-11-21

**Authors:** Die Hu, Yongli Zhang, Defei Liu, Depei Wang, Chaoguang Tian

**Affiliations:** ^1^College of Biotechnology, Tianjin University of Science and Technology, Tianjin, China; ^2^Key Laboratory of Systems Microbial Biotechnology, Tianjin Institute of Industrial Biotechnology, Chinese Academy of Sciences, Tianjin, China; ^3^National Technology Innovation Center of Synthetic Biology, Tianjin, China

**Keywords:** filamentous fungi, 6-phosphofructo-2-kinase/fructose-2,6-biphosphatase, *Myceliophthora thermophila*, metabolic regulation, glycolysis, metabolic engineering

## Abstract

The key enzyme 6-phosphofructo-2-kinase (PFK2)/fructose-2,6-bisphosphatase (FBPase-2) is responsible for regulating the rates of glycolysis and gluconeogenesis in eukaryotes. However, its functions and mechanisms in filamentous fungi remain largely enigmatic. In this study, we systematically investigated the function of this enzyme in *Myceliophthora thermophila*, a thermophilic filamentous fungus with great capacity to produce industrial enzymes and organic acids. Our results showed that the *M. thermophila* genome encodes three isomers, all with the PFK2/FBPase-2 structure: *pfk2-a, pfk2-b*, and *pfk2-c*. Overexpression of each gene revealed that endogenous expression of *pfk2-c* (PFK2 activity) promoted glucose metabolism, while overexpression of *pfk2-a* (FBPase-2 activity) inhibited strain growth. Using knockouts, we found that each gene was individually non-essential, but the triple knockout led to significantly slower growth compared with the wild-type strain. Only the *pfk2-a* single knockout exhibited 22.15% faster sugar metabolism, exerted through activation of 6-phosphofructo-1-kinase (PFK1), thereby significantly promoting glycolysis and the tricarboxylic acid cycle. The FBPase-2 deletion mutant strain also exhibited overflow metabolism, and knocking out *pfk2-a* was proved to be able to improve the production and synthesis rate of various metabolites, such as glycerol and malate. This is the first study to systematically investigate the function of PFK2/FBPase-2 in a thermophilic fungus, providing an effective target for metabolic engineering in filamentous fungi.

## Introduction

In eukaryotic organisms, the glycolytic pathway is tightly regulated to maintain metabolic homeostasis. Among the most important regulators of this pathway is fructose 2,6-bisphosphate (fru-2,6-P_2_). The fru-2,6-P_2_ metabolite has been found in almost all eukaryotes, throughout the animal, plant and fungi kingdoms, but not in bacteria (with the exception of *Desulfovibrio desulfuricans*) (Rider et al., 2004). In most eukaryotic cells, fru-2,6-P_2_ is (1) a potent positive allosteric, acting at submicromolar concentrations; (2) an effector of 6-phosphofructo-1-kinase (PFK1), and thus a stimulator of glycolysis; and (3) a possible inhibitor of gluconeogenesis, acting through inhibition of fructose-1,6-bisphosphatase (FBPase-1) in some cell types (Michels and Rigden, 2006).

Synthesis of fru-2,6-P_2_ from fructose-6-phosphate (F6P) and ATP is catalyzed by 6-phosphofructo-2-kinase (PFK2), and its hydrolysis to fructose-6-phosphate and inorganic phosphate Pi is catalyzed by fructose-2,6-bisphosphatase (FBPase-2) (Pilkis, 1995; Okar et al., 2001). Interestingly, the PFK2 and FBPase-2 reactions are catalyzed on the same polypeptide, which functions within a homodimeric or tetrameric protein (Okar et al., 2001; Nielsen et al., 2004). The PFK2 reaction is catalyzed in the N-terminal half of the enzyme subunit, whereas the FBPase-2 reaction is catalyzed in the C-terminal half. Similar to fru-2,6-P_2_, PFK2/FBPase-2 isoenzymes have been identified in all major eukaryotic taxa (Rider et al., 2004).

In mammals, there are four PFK2/FBPase-2 isoenzymes, one each in the liver, heart, brain (or placenta) and testes, which differ by the sequence of their bifunctional catalytic core (Rider et al., 2004). These isoenzymes are homodimers, arranged head-to-head with the PFK2 domains making contact (Rider et al., 2004). In mammalian tissues, fru-2,6-P_2_ acts as a glucose signal to stimulate glycolysis when glucose is available. Expression of the PFK2/FBPase-2 coding genes is regulated by insulin and glucagon to maintain glucose homeostasis (Raben, 2013). Apart from the functions of PFK2/FBPase-2 in normal cells, by modulating the levels of fru-2,6-P_2_, these enzymes are crucial players in regulating the metabolic activity and anaerobic adaptation of cancer cells (Ros and Schulze, 2013; Chesney et al., 2014; Icard et al., 2022).

In plants, a bifunctional PFK2/FBPase-2 enzyme has also been identified, but molecular data suggest that it is encoded by a single gene (Nielsen et al., 2004). Different from animals, PFK2 purified from spinach leaves was shown to function as a tetramer (Larondelle et al., 1986), an observation that has been corroborated by heterologous expression (Markham and Kruger, 2002). In plants, fru-2,6-P_2_ has a key function in coordinating the photosynthetic carbon flux into sucrose and starch biosynthesis (Nielsen et al., 2004; Lee et al., 2006).

In *Saccharomyces cerevisiae*, three isoenzymes have been found that are homologous to the mammalian and plant bifunctional enzymes. Yeast PFK26 and FBP26 share the PFK2/FBPase-2 structure, while yeast PFK27 only contains a PFK2 domain and a highly aberrant C-terminal domain (Kretschmer et al., 1987; Rider et al., 2004). However, in each of the yeast isoforms, one activity has been lost as a result of substitutions and deletions. *PFK27* and *PFK26* each encode an isozyme of PFK2, and *FBP26* encodes FBPase-2. PFK27 synthesis is induced by fermentable carbon sources, while PFK26 is activated by protein kinase A phosphorylation (Kretschmer and Fraenkel, 1991). It is unclear why the bifunctional PFK2/FBPase-2 might have evolved to function as multiple mono-functional enzymes in *S. cerevisiae*.

The phylum Ascomycota (except *Schizosaccharomyces pombe*) contains three homologous isoforms of PFK2 (Sellem et al., 2019), but there is little information about their functions. To investigate the role of PFK2 in Ascomycota, we systematically investigated the functions of these genes in *Myceliophthora thermophila*. This typical filamentous fungus has great capacity for biomass degradation and features multiple characteristics of industrial interest, including high-temperature fermentation, substantial protein secretion capacity, and fast growth on cellulose (Bhat and Maheshwari, 1987; Karnaouri et al., 2014). In recent years, *M. thermophila* has been developed as a platform for industrial enzyme and organic acid production from plant biomass (Visser et al., 2011; Li et al., 2019). The genome of the *M. thermophila* wild-type (WT) strain (American Type Culture Collection [ATCC] 42464) has been completely sequenced and annotated (Berka et al., 2011). The development and usage of tools for genetic manipulation and CRISPR-Cas9-based genome editing have facilitated the recent systematic examination and identification of gene functions in this fungus (Xu et al., 2015; Liu et al., 2017).

We have conducted the first systematic study of PFK2/FBPase-2 function in Ascomycota, overexpressing and knocking out all three coding genes (*pfk2-a*[Mycth_71484], *pfk2-b*[Mycth_2301421], and *pfk2-c*[Mycth_2312639]) individually and in combination. For mutants with distinct phenotypes, transcriptomes and changes in central carbon metabolism were characterized and analyzed. Finally, we tested the feasibility of increasing glycolytic flux to improve the production of various metabolite compounds via knockout of the FBPase-2 gene. Our results indicate that manipulating the regulation of this gene has good application potential for metabolic engineering.

## Materials and methods

### Strains, media, and culture conditions

*Myceliophthora thermophila* ATCC 42464 was obtained from the ATCC. All strains used in this study are listed in Supplementary Table 1. The strains were grown on Vogel’s minimal medium supplemented with 2% (w/v) sucrose (MM medium) at 35°C for 10–15 days to obtain mature conidia. Antibiotics were added when needed to screen for transformants. For flask cultures, conidia of *M. thermophila* were inoculated into 100 mL fermentation medium to a final concentration of 2 × 10^5^ conidia/mL in a 250-mL Erlenmeyer flask. The fermentation medium (per liter) consisted of 75 g glucose, 10 g yeast extract, 0.15 g KH_2_PO_4_, 0.15 g K_2_HPO_4_, 0.15 g MgSO_4_⋅7H_2_O, 0.1 g CaCl_2_⋅2H_2_O, 1 mL biotin (0.1 g/L), and 1 mL trace elements (Vogel’s salts). *Escherichia coli* strains DH5α and BL21(DE3) were used for plasmid amplification and protein expression, respectively, and were cultured in Luria–Bertani (LB) medium supplemented with antibiotics as necessary.

### Construction of expression and deletion plasmids

The primers used in this study are listed in Supplementary Table 2.

For constructing the vector-carrying donor DNA for disrupting *pfk2*, the 5′- and 3′-flanking fragments of *pfk2* were amplified from *M. thermophila* genome. PtrpC-bar from the p0380-bar plasmid and PtrpC-neo from the p0380-neo plasmid were each cloned using primers and fused with *pfk2*-up and *pfk2*-down, respectively. The fragments 5′-*pfk2-a*-up-Bar-down-3′ and 5′-*pfk2-b/c*-up-Neo-down-3′ were created by overlapping PCR and cloned into the pJET1.2/blunt cloning vector.

To select for specific single-guided RNAs (sgRNAs) targeting *pfk2-a*, *pfk2-b*, and *pfk2-c*, we used the sgRNA-Cas9 tool to identify sgRNA target sites with high scores (Shengsong et al., 2014). Then, a target-directed *M. thermophila* U6 promoter-driven sgRNA was created by overlapping PCR and cloned into the pJET1.2/blunt cloning vector, giving the corresponding plasmids U6-*pfk2-a*-sgRNA, U6- *pfk2-b*-sgRNA, and U6- *pfk2-c*-sgRNA. The Cas9-expression PCR cassette Ptef1-Cas9-TtprC was amplified using Ptef-cas-F/T*tprC*-cas-R from the plasmid p0380-bar-P*tef1*-Cas9-T*tprC* (Liu et al., 2017).

For overexpression of *pfk2-a/b/c*, fragments of *pfk2-a/b/c* were amplified from *M. thermophila* DNA using the primers OE*pfk2-a/b/c*-F and OE*pfk2-a/b/c*-R and assembled into *Bcu*I/*Bam*HI-digested pAN52-MtP*gpdA*-T*tprC*-Neo using the NEB Gibson assembly kit (New England Biolabs, Ipswich, MA, USA). The open reading frame of *pfk1* of *Neurospora crassa* (NCU202198) was amplified from cDNA of *N. crassa* using the primers Ncpfk1-F/Ncpfk1-R and assembled into *Bcu*I/*Bam*HI-digested pAN52-MtP*gpdA*-T*tprC* using the NEB Gibson assembly kit. The pAN52-P*trpC*-*hph*-P*tef*-*Aomae*-P*AngpdA*-*Aopyc* plasmid construction was the same as that described previously (Li et al., 2019). *GsalaD* from *Geobacillus stearothermophilus* and *Scgpd1* from *S. cerevisiae* were cloned into pAN52-P*gpdA*-Neo to generate the plasmids pAN52-P*gpdA*-*GsalaD*-Neo and pAN52-P*gpdA*-*Scgpd1*-Neo, respectively, using the NEB Gibson assembly kit.

All vectors were constructed using *E. coli* DH5α and all constructed plasmids were verified by DNA sequencing.

### Transformation of *Myceliophthora thermophila* protoplasts

Transformation of *M. thermophila* protoplasts was performed using a previously described procedure (Liu et al., 2017). For target gene expression, 10 μg plasmid was added to the fungal protoplasts (Wang et al., 2015). For gene disruption, sgRNA, the donor expression cassette for disruption, and the Cas9-expression PCR cassette were mixed and co-transformed into the WT strain. Colonies were screened for bar or neo gene resistance using phosphinothricin (150 μg/mL) or geneticin (80 μg/mL), followed by sequential identification via PCR with paired primers. Gene disruption by CRISPR/Cas9 was performed as described previously (Liu et al., 2017).

### Growth test and dry weight assay

To compare growth of WT and other strains in solid medium, conidia of *M. thermophila* were collected from each strain, filtered and adjusted to 2.5 × 10^7^ conidia/mL. A 2-μL aliquot of the suspension was plated on fermentation medium supplemented with 7.5% glucose at 45°C for 3 days. To evaluate glucose consumption in each strain, 100 mL of medium was inoculated with mature spores (2.5 × 10^5^ spores/mL) and batch-cultured in a 250-mL Erlenmeyer flask. The culture was incubated at 45°C on a rotary shaker at 150 rpm. Samples (5 mL) were taken at different intervals. The fungal biomass dry weights were estimated at 8 days after inoculation of the Erlenmeyer flask. Fungal mycelial biomass was then harvested, dried, and weighed.

### High-performance liquid chromatography analysis of extracellular metabolites

The cultures were centrifuged and the resulting supernatants were filtered through a 0.22-μm filter (Corning, NY, USA). HPLC was used to detect and quantify the glucose, glycerin, and malate acid using an e2695 instrument (Waters, Manchester, UK) and an Aminex HPX-87H column (Bio-Rad, Hercules, CA, USA) at 45°C. The mobile phase (5 mM H_2_SO_4_) was set at a flow rate of 0.5 mL min^–1^ and the compounds detected with a Waters 410 refractive index detector (Waters Corporation, Milford, MA, USA).

### Expression and purification of PFK2/FBPase-2

*Escherichia coli* Rosetta (DE3) transformed with PFK2A-pET32a was grown in LB at 37°C to an optical density at 600 nm of 0.6, and then protein expression was induced with 1.4 mM isopropyl β-D-thiogalactoside (IPTG) at 16°C for 15 h. Expression of PFK2B/C-pET28a was induced with 0.4 mM IPTG. Cells were lysed at 4°C using Scientz-IID ultrasonic processors (SCIENTZ, Zhejiang, China) in phosphate-buffered saline (PBS), and the cell debris was cleared by centrifugation at 4°C. Cleared lysates were applied to a 1-mL His-Trap column (GE Healthcare, Pittsburgh, USA) and purified by an AKTA avant150 (GE Healthcare) with a 50–500 mM gradient of imidazole buffer. The purified protein was detected following sodium dodecyl sulfate-polyacrylamide gel electrophoresis (SDS-PAGE). Protein concentrations were determined using a Bio-Rad Protein Assay kit (Bio-Rad, Hercules, CA, USA).

### Enzyme activity assays

PFK2 activity was measured as described previously except that the protein concentration used was 15 μg (Kurland et al., 2000; Manes and El-Maghrabi, 2005). The fru-2,6-P_2_ produced was then quantified by measuring potato inorganic pyrophosphate-PFK activation spectrophotometrically as described previously, except that the reaction was scaled down for use on a 96-well plate (Schaftingen et al., 1982). FBPase-2 activity was measured by incubating 15 μg protein for 60 min at 30°C in assay buffer at pH 7 with 1 mM ATP, 2 mM MgCl_2_, 5 mM KPi, and 50 μM fru-2,6-P_2_. Reactions were halted by adding NaOH to a final concentration of 100 mM (Kurland et al., 2000; Manes and El-Maghrabi, 2005). Concentrations of the fru-2,6-P_2_ product were determined using the liquid chromatography-tandem mass spectrometry (LC–MS/MS) platform.

Activity of PFK1 in the *M. thermophila* mycelial cultures was determined using the PFK1 Assay Kit (Solarbio, Beijing, China).

### RNA extraction, sequencing, and data analysis

*Myceliophthora thermophila* strains were used to inoculate fermentation medium containing a 7.5% carbon source and cultured at 45°C. Mycelia were collected at 2 and 4 days of culture, immediately homogenized in liquid nitrogen, and then stored at −80°C. Total RNA was extracted as described previously (Xu et al., 2018). Total RNA was extracted from mycelia through mechanical disruption with mortar and pestle, digested with DNase1, and purified using the Qiagen RNeasy Mini kit (Qiagen, Hilden, Germany). A cDNA library was then synthesized and sequenced using the DNBSEQ platform at BGI (Shenzhen, China). All data in this study were generated by sequencing two independent biological replicates.

### Quantitative real-time PCR

The qRT-PCR analysis was performed using the iScript cDNA Synthesis Kit, the SYBR Green Realtime PCR Master Mix (TOYOBO, Osaka, Japan), and the CFX96 Real-Time PCR Detection System (Bio-Rad). The qRT-PCR was performed as follows: 95°C for 30 s, 40 cycles of 95°C for 30 s, 55°C for 30 s, and 72°C for 30 s. A melt curve analysis was performed at the end of each run from 55 to 95°C with a ramp speed of 0.5°C to ensure specific sequence amplification of all primers and only one melting temperature on the melting curve. Each reaction was performed in quadruplicate. The actin gene (Mycth_2314852) was used as an endogenous control for all experiments. The transcription level of each gene was estimated using the 2^–ΔΔ*Ct*^ method (Livak and Schmittgen, 2013). The ratio of the transcription level in the mutant to that in the WT control was calculated as the relative transcription level.

### Liquid chromatography tandem mass spectrometry analysis of intracellular metabolites

The samples for intracellular metabolite profiling were prepared and analyzed using the standardized and improved LC–MS/MS metabolomics methodology (Xiaomei et al., 2019). Briefly, 20 mL (±0.5 mL) of *M. thermophila* mycelial culture was fast filtered through a –20°C precooled vacuum filter. After washing with 25 mL precooled PBS buffer, the mycelial samples were flash frozen into liquid nitrogen. After pulverization by pestle and mortar in liquid nitrogen, about 0.1 g (±0.01 g) of sample was suspended in 1 mL precooled 50% methanol and incubated at 4°C for 15 min. Exact weight was determined by weighing each tube before and after adding the samples. After placing on ice for 5 min, the mixture was centrifuged at 12,000 g at 4°C for 15 min to remove cell debris, and the supernatant was collected into a precooled tube (Zhang et al., 2020). The supernatant was then allowed to evaporate under vacuum using a centrifugal vacuum concentrator. The dried metabolites were dissolved in 20 μL Milli-Q water and then analyzed by the LC–MS/MS platform comprising an ultra-performance liquid chromatography (UPLC) 30A system (Shimadzu, Kyoto, Japan) and a TripleTOF™ 6600 mass spectrometer (Applied Biosystem Sciex, Framingham, MA, USA), as described previously. The LC–MS/MS data were normalized by sample weight, and then metabolites involved in central metabolism were identified using the protocol described (Xiaomei et al., 2019).

### Statistical significance tests

One-tailed homoscedastic (equal variance) *t*-test was used for adjusting statistical significance. n.s., no statistical significance; *p < 0.05; **p < 0.01; and ***p < 0.001.

## Results

### PFK2/FBPase-2 is a monofunctional enzyme in *Myceliophthora thermophila*

**FIGURE 1:**
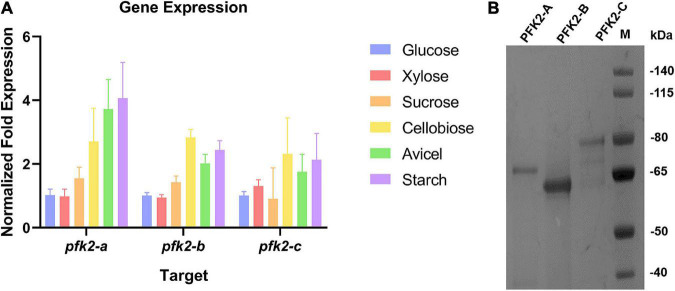
The qRT-PCR analysis of gene expression and protein purification of PFK2/FBPase-2 in *Myceliophthora thermophila*. **(A)** Expression levels of the three coding genes in *M. thermophila* after 2 days of culture in media containing glucose, xylose, sucrose, cellobiose, Avicel and starch; actin was used as the reference gene. **(B)** Coomassie blue staining of purified PFK2-A/B/C proteins following SDS-PAGE. PFK2-A/B/C were expressed in *Escherichia coli* and purified by Ni chelate affinity chromatography. Molecular weight markers in kDa are shown at the right.

In Ascomycetes, most species have three isoforms of PFK2/FBPase-2 (Sellem et al., 2019), as does *M. thermophila*. These three isoforms are encoded by Mycth_71484 (*pfk2-a*), Mycth_2301421 (*pfk2-b*), and Mycth_2312639 (*pfk2-c*). Sequence analysis of the corresponding proteins, as shown in Supplementary Figure 1, revealed that PFK2-A, PFK2-B and PFK2-C each contain a PFK2 domain and a FBPase-2 domain. Next, we analyzed the expression patterns of the three genes under multiple carbon source conditions. Generally, the expression level of *pfk2-a* was the highest among the three genes, while those of *pfk2-b* and *pfk2-c* were comparable, consistent with previous transcriptome results (Li et al., 2019, 2020). Comparing different carbon sources, the gene expression levels were similar under glucose, xylose and sucrose, and lower than those under cellobiose, avicel, and starch (Figure 1A).

To gain further insights into the functions of PFK2/FBPase-2 in this thermophilic fungus, cDNAs encoding the proteins were expressed in the *E. coli* strain BL21(DE3) (Figure 1B). The results of enzyme activity determination are summarized in Table 1. Genes *pfk2-a* and *pfk2-c* were shown to encode monofunctional FBPase-2 and monofunctional PFK2 activities, respectively. However, for *pfk2-b*, neither FBPase-2 nor PFK2 activity was detected, even at high concentration. These results indicated that PFK2/FBPase-2 is encoded by monofunctional genes in filamentous fungi, which is different from mammals and plants. Separated FBPase-2 and PFK2 activities were also found in *S. cerevisiae*, which has been attributed to crucial substitutions or major deletions in one of the two protein domains that occurred during evolution, rendering them monofunctional.

**TABLE 1:** Assays of 6-phosphofructo-kinase-2 (PFK2) and fructose-2,6-bisphosphatase (FBPase-2) enzymatic activity of *Myceliophthora thermophila* PFK2-A/B/C proteins expressed in *Escherichia coli*.

	V_*max*_^a^ (mkat/mg prot)	PFK2		FBPase-2
			
		K_M_ (ATP) (mM)	K_M_ (F6P) (mM)	K_M_ (F-2,6-P_2_) (mM)
PFK2-A	197.53 ± 5.42	NA^c^	NA	3.81 ± 0.22
PFK2-B	ND^b^	——^d^	——	——
PFK2-C	3.26 ± 0.14	0.94 ± 0.15	1.43 ± 0.09	NA

Enzymes were expressed in *E. coli*, purified and assayed as described in the “Materials and methods” section. Values represent means ± standard errors for three determinations of V_max_ and K_M_ for each enzyme; ^a^V_max_ of PFK2-A for FBPase-2 activity and V_max_ of PFK2-C for PFK2 activity; ^b^ND, not determined; ^c^NA, not applicable; ^d^Absence of detectable PFK2-B catalytic activity, even in the presence of 100 μg substrate. The K_M_ of PFK2-B could not be calculated because of its low activity.

### Different PFK2/FBPase-2 mutants have different phenotypes

**FIGURE 2:**
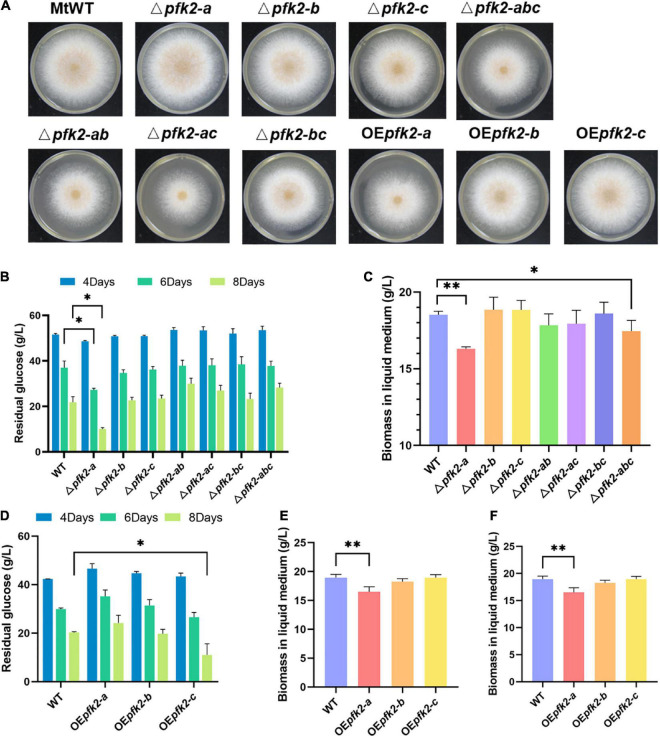
Phenotypic analysis of the *pfk2* knockout mutants and overexpression strains. **(A)** Growth comparison of *Myceliophthora thermophila* WT, knockout mutant and overexpression strains after 3 days of culture on solid plates. Comparison of glucose consumption **(B)** and biomass in liquid medium **(C)** between WT and the mutant strains after 8 days of culture in liquid media. Comparison of glucose consumption **(D)**, biomass in liquid medium **(E)**, and glucose consumption/unit biomass **(F)** between the WT and overexpression strains after 8 days of culture in liquid media. Values and error bars represent means and standard deviations of independent triplicate experiments, respectively. *p < 0.05; **p < 0.01.

To learn more about the function of PFK2/FBPase-2, single, double, and triple gene deletion knockouts of *pfk2-a/b/c* were performed using the CRISPR/Cas9 system in WT. Each of the single-knockout mutants were able to survive, indicating that all three genes were non-essential genes, but the phenotypes of these knockout strains varied. On solid media, the Δ*pfk2-a* and Δ*pfk-b* mutants grew as fast as WT, but the growth of Δ*pfk2-c* was slower than WT which was consistent with its PFK2 function. All of the double-knockout mutants grew slower than the WT strain (Figure 2A), indicating that their combined effect is to promote growth. Despite the observation that *pfk-b* did not encode a protein with detectable enzymatic activity, the strains carrying a double knockout of *pfk-a* and *pfk-b* grew more slowly than the Δ*pfk-a* strain, suggesting that PFK2-B may indeed have PFK2 activity. Complete deletion of all three genes also resulted in slower growth compared with the WT strain. To further analyze the effect of each gene on growth, the same comparisons were made in liquid media. Interestingly, we observed that Δ*pfk2-a* had a lower biomass, but a higher glucose consumption rate, than WT (Figures 2B,C). The biomass of Δ*pfk2-a* decreased from 18.17 to 16.30 g/L of dry cell weight, 10.3% lower than that of WT, and glucose consumption increased to a rate 22.15% higher than that of WT. Furthermore, the glucose consumption rate/dry cell weight was 36.18% higher than that of WT. Taken together, the deletion of *pfk2-a* appeared to be beneficial for improving the rate of glucose metabolism. Another notable observation of the Δ*pfk2-abc* mutant was that, compared with the WT strain, the biomass was reduced and the glucose consumption rate was 12.11% lower (Figures 2B,C). This was consistent with the phenotype on solid media, indicating that simultaneous deletion of all three genes affects the growth and metabolism of *M. thermophila*.

We further investigated the functions of all three genes using an overexpression system wherein *pfk2-a, pfk2-b* and *pfk2-c* were placed under the strong constitutive promoter of *gpdA* in WT. No obvious growth differences were seen between the *pfk2-b* and *pfk2-c* overexpression (OE*pfk2-b* and OE*pfk2-c*) and WT strains (Figure 2D). However, the *pfk2-a* overexpression strain (OE*pfk2-a*) grew more slowly than WT on solid media plates, and had 12.88% less biomass than WT in liquid media (Figures 2D,E). This was likely due to overexpression of FBPase-2 activity, which catalyzes the degradation of fru-2,6-P_2_, thereby reducing the rate of glycolysis, this might have contribution to the decreasing of biomass production. By contrast, the *pfk2-c* overexpression strain showed no difference in biomass compared to WT, but the glucose consumption rate/unit biomass was 15.5% higher than that of WT in liquid medium (Figure 2F). This was consistent with its PFK2 activity, which catalyzes the synthesis of fru-2,6-P_2_, thereby enhancing the rate of glycolysis. Unlike the other two genes, the *pfk2-b* overexpression strain showed no significant differences from the WT strain in either liquid or solid media, possibly because it does not possess substantial PFK2 or FBPase-2 activity.

### Metabolome analysis of the Δ*pfk2-a* and Δ*pfk2*-*abc* mutants under glucose conditions

Among the various *pfk2-a/b/c* mutants, the Δ*pfk2-a* and Δ*pfk2-abc* strains showed significant phenotypic changes. Compared with WT, Δ*pfk2-a* exhibited a significantly accelerated rate of glucose consumption, while Δ*pfk2-abc* showed a lower rate. This intriguing result prompted us to undertake a quantitative examination of the changes in intracellular metabolism in the mutants. The *pfk2-a* gene, which encodes FBPase-2, plays an essential role in fru-2,6-P_2_ degradation; indeed, the triple-knockout mutant completely lost the ability to synthesize and degrade fru-2,6-P_2_. Samples were taken for metabolomics analysis during fermentation at days 2–4, and the relative level of each metabolite in the Δ*pfk2-a* and Δ*pfk2-abc* strains was calculated by dividing its normalized peak area by that in the WT strain.

**FIGURE 3:**
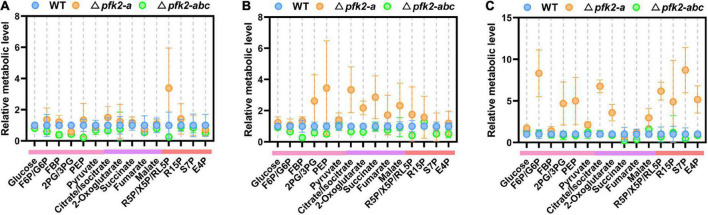
Intracellular metabolite profiling of intermediates involved in central metabolic pathways in the WT, Δ*pfk2-a*, and Δ*pfk2-abc* strains. Metabolites from the EMP pathway (pink lines), TCA cycle (purple lines), and PPP (red lines) on days 2, 3, and 4 are shown in panels **(A–C)**. The concentrations of extracted intracellular intermediates were measured by LC–MS/MS, and the relative level of each metabolite in the Δ*pfk2-a* and Δ*pfk2-abc* strains was calculated by dividing its normalized peak area by that in the WT strain. Values and error bars represent means and standard deviations of independent triplicate experiments, respectively. F6P, fructose-6-phosphate; FBP, fructose-1,6-diphosphate; 2PG, 2-phosphoglycerate; 3PG, 3-phosphoglycerate; PEP, phosphoenolpyruvate; R5P, ribose-5-phosphate; X5P, xylulose-5-phosphate; RL5P, ribulose-5-phosphate; R15P, ribulose-1,5-bisphosphate; S7P, sedoheptulose-7-phosphate; E4P, erythrose-4-phosphate.

On day 2, there were no significant differences in intracellular metabolite content among the three strains (except for some pentose in the pentose phosphate pathway [PPP] of Δ*pfk2-a*) (Figure 3A). On day 3, the concentrations of metabolites in the Embden Meyerhof Parnas (EMP) pathway in Δ*pfk2-a* were higher than those in WT. These metabolites included fructose-1,6-diphosphate (FBP), 2/3-phosphoglycerate (2PG/3PG), and especially phosphoenolpyruvate (PEP), whose concentration was 3.45-fold higher in Δ*pfk2-a* than in WT. Among the tricarboxylic acid (TCA) cycle intermediates, citrate/isocitrate, 2-oxoglutarate, succinate, fumarate, and malate were 3. 34-, 2. 18-, 2. 86-, 1. 72-, and 2.32-fold higher in Δ*pfk2-a* than in WT, respectively. Intermediates of the PPP were also higher in Δ*pfk2-a* than in WT (Figure 3B). The content of most amino acids was also higher in Δ*pfk2-a* compared with WT (Supplementary Figure 2). The above results showed that, in the absence of FBPase-2 activity, the degradation of fru-2,6-P_2_ is reduced, thereby promoting glycolytic metabolism, which in turn promotes the metabolism of TCA. We suspect that the increase in amino acid content may have been due to the vigorous glycolysis and metabolism of TCA, which requires more protein synthesis in cells. The synthesis of amino acids promotes the consumption of intermediate metabolites and NADPH oxidation in the PPP and, coupled with the enhancement in TCA metabolism, promotes the metabolism of the PPP. Similar to day 3, increased metabolism of the above pathways was also observed in Δ*pfk2-a* on day 4 (Figure 3C).

By contrast, the levels of most intracellular intermediates of the PPP, EMP pathway and TCA cycle were lower in Δ*pfk2-abc* mutants than in WT (Figures 3A–C), consistent with the rate of glucose consumption. In the triple knockout, the strain lost the ability to synthesize fru-2,6-P_2_, and without the activation of fru-2,6-P_2_, the rate of glycolysis was reduced and the metabolism of other downstream pathways also slowed down. These results indicated that PFK2/FBPase-2 plays an important role in the metabolism and growth of *M. thermophila*, helping fungi better compete for nutrients and grow faster in nutrient-rich environments. Complete deletion of its coding genes reduces the metabolism of carbon and growth rate.

### PFK2 promotes central carbon metabolism by activating PFK1

**FIGURE 4:**
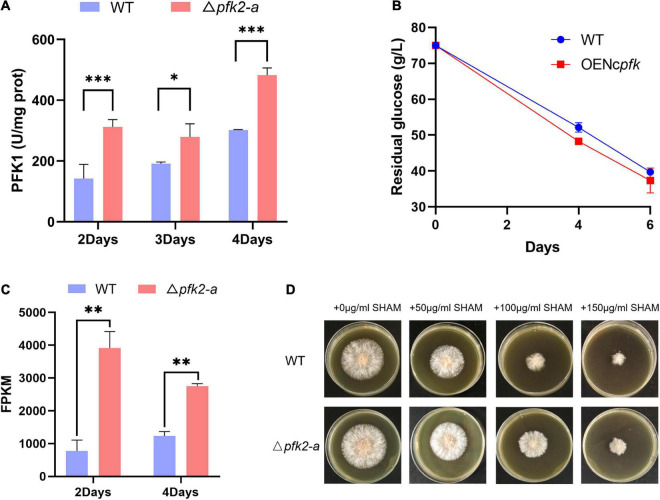
Regulatory role of *pfk2-a* in the metabolism of *Myceliophthora thermophila*. **(A)** PFK1 activity of WT and Δ*pfk2-a* mutants at 2–4 days of mycelial culture. **(B)** Glucose consumption of the WT and OE*Ncpfk* strains in glucose medium. **(C)** Comparison of the fragments per kilobase of exon model per million mapped fragments (FPKM) of *aox* in the WT and Δ*pfk2-a* mutant strains at 2 and 4 days. **(D)** Comparative expression of AOX in the WT and Δ*pfk2-a* mutant strains derived from testing the resistance to SHAM, a specific inhibitor of the alternative respiratory pathway. Stronger SHAM resistance was considered an indicator of higher AOX expression. *p < 0.05; **p < 0.01 and ***p < 0.001.

To determine whether PFK2 in filamentous fungi promotes glycolysis by activating PFK1, as previous research has shown in mammals and yeast (Bolesm et al., 2010; Ros and Schulze, 2013), we measured the enzymatic activities of PFK1 in the WT and mutant strains. We found that the PFK1 activity in Δ*pfk2-a* was significantly higher than that in WT, increasing by 119, 46, and 60% on days 2, 3, and 4, respectively (Figure 4A). We speculate that the deletion of *pfk2-a* promotes glycolytic metabolism in response to the loss of FBPase-2 activity; with only PFK2 activity remaining, PFK1 is activated.

To verify the above speculation, we overexpressed *pfk1* from *N. crassa* in WT (OE*Ncpfk*), and found that the overexpression strain had a faster glucose consumption rate than the WT strain (Figure 4B). These results indicated that increasing PFK1 activity promoted glucose consumption in *M. thermophila*, further confirming that the promotional effect of Δ*pfk2-a* is exerted via activation of PFK1.

One response to rapid metabolism is a significant increase in the expression of alternative oxidase (AOX) in the mitochondrial electron transport chain (Figure 4C). This relaxes the tension in the highly coupled electron transport process in mitochondria, thus providing and maintaining metabolic homeostasis (Saha et al., 2016). We further estimated the amount of AOX from growth resistance of mycelial cultures to salicylhydroxamic acid (SHAM), a specific inhibitor of the alternative respiratory pathway. The correlation between a strong constitutive induction of the alternative pathway and the suppressor effect was also assessed at the protein level showing an increased resistance to SHAM. The growth on solid media shown in Figure 4D demonstrated that knockout of *pfk2-a* (encoding FBPase-2) led to higher expression of *aox*.

### Knockout of *pfk2-a* increases the production of multiple metabolites

**FIGURE 5:**
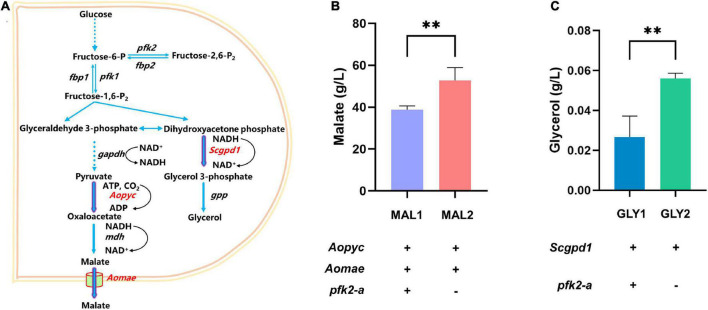
Effects of the *pfk2-a* knockout on the promotion of metabolite production. **(A)** Schematic illustration of the malate and glycerol synthesis route in *Myceliophthora thermophila*. **(B)** Malate production with and without the *pfk2-a* deletion in the MAL1 and MAL2 strains during 6 days of shake flask fermentation. **(C)** Glycerol production with and without the *pfk2-a* deletion in the GLY1 and GLY2 strains during 6 days of shake flask fermentation. Values and error bars represent means and standard deviations of independent triplicate experiments, respectively. **p < 0.01.

The above results showed that knockout of *pfk2-a* promoted the metabolic rate. To verify whether knocking out *pfk2-a* could improve metabolites production, the production of metabolites was increased in the Δ*pfk2-a* strain, the glycerol and malate were randomly selected for validation (Figure 5A). We created a *pfk2-a* knockout in a malate-producing strain that was engineered via heterologous overexpression of the malic acid exporter (*mae*) and pyruvate carboxylase (*pyc*) gene from *Aspergillus oryzae* (strain MAL1). The resulting strain, MAL2, produced 52.834 g/L malate acid, a 36.2% increase over that of its parental strain (Figure 5B). Similarly, we introduced glyceraldehyde-3-phosphate dehydrogenase (*gpd1*) from *S. cerevisiae* (strain GLY1) driven by the strong constitutive promoter of *gpdA* into WT, to increase glycerol production. We then created a *pfk2-a* knockout in the overexpression strain (GLY2) using the CRISPR/Cas9 system. As shown in Figure 5C, the titer of glycerol increased from 0.027 g/L in GLY1 strain to 0.056 g/L in the *pfk2-a* deletion mutant (strain GLY2), a 107% difference. These data suggested that knockout of *pfk2-a* may be developed as a strategy to engineer increases in the yield of metabolites and rate of metabolism in *M. thermophila*.

## Discussion

The PFK2/FBPase-2 protein contains important regulatory enzymatic activities in the glycolytic pathway and is present in all major eukaryotic taxa. However, unlike the well-studied animal and plant PFK2/FBPase-2 enzymes, few studies have investigated the corresponding functions in the filamentous fungi. We chose to characterize the functions of *pfk2-a/b/c* in the typical filamentous fungus and industrial microorganism *M. thermophila*, and then investigate the potential for regulating these genes to improve metabolite production. The results of *in vitro* activity assays showed that *M. thermophila* PFK2-A and PFK2-C were monofunctional enzymes, and that PFK2-B had no detectable catalytic activity. While overexpression of PFK2-C and knockout of the PFK2-A gene both promoted glucose metabolism, the *pfk2-a* deletion was particularly effective. Metabolic changes in the Δ*pfk2-a* strain were due to the activation of PFK1, which promoted central carbon metabolism overall, including the TCA cycle. Additionally, we demonstrated that the strategy of knocking out *pfk2-a* increased the production of multiple metabolites, including malate and glycerol.

Regarding the domain organization of the subunit sequences of the PFK2/FBPase-2 isoenzymes, we searched for orthologs of *M. thermophila* using National Center for Biotechnology Information resource. Among all the other fungi tested, we found one ortholog of each of the three proteins. We attributed the letter “a” to *pfk2-a* orthologs (sub family 1), “b” to *pfk2-b* orthologs (sub family 2), and “c” to *pfk2-c* orthologs (sub family 3). To explore the phylogenetic relationships among these different proteins, we generated a maximum likelihood phylogenetic tree, shown in Supplementary Figure 3. Except for *S. cerevisiae* PFK27, histidine in the RHG motif in sub family 2 were replaced by alanine (Supplementary Figure 4), which eliminated the phosphatase activity because histidine is the phosphorus receptor in the double phosphatase reaction. Sequence alignment revealed conservation of key amino acids in the first histone domain, but histidine was substituted by alanine in the RHG motif of *pfk2-b*, indicating that *M. thermophila pfk2-b* may not have phosphatase activity. However, the Δ*pfk2-ab* double-mutant strain grew more slowly than the Δ*pfk2-a* single-mutant strain, suggesting that *pfk2-b* may encode a PFK2 homolog with a low level of catalytic activity. The PFK2 domain is structurally similar to the adenylate kinase family, while the FBPase-2 domain is related to the phosphoglycerate mutase and histidine acid phosphatase families, which share a common centrally involved RHG motif (Michels and Rigden, 2006; Sellem et al., 2019). Mutation of histidine in the liver and testis isoenzymes in rats, and in PFK26 in yeast, confirmed the importance of the covalent phosphohistidine intermediate in the RHG motif for FBPase-2 activity.

The *in vivo* results showed that both overexpression of PFK2 (*pfk2-c*) and knockout of FBPase-2 (*pfk2-a*) promoted the metabolism of *M. thermophila*, particularly the FBPase-2 knockout. The *pfk2-a* deletion mutant significantly accelerated the rate of glucose utilization. Similar in mammals and *S. cerevisiae*, fru-2,6-P_2_ is an allosteric activator of PFK1, thus a stimulator of glycolysis (Lee et al., 2018; Tanner et al., 2018). The PFK1 activity assay and overexpression of *N. crassa* PFK1 showed that the altered metabolism of the Δ*pfk2-a* strain was due to the increased activity of PFK1, which in turn promoted the metabolism of the EMP pathway and TCA cycle.

The observation that overexpression of PFK2 promoted the glucose metabolic rate indicated that the role of PFK2 is to promote glycolysis. Knockout of *pfk2-a* significantly accelerated the metabolic rate of *M. thermophila*, indicating that FBPase-2 is normally used to prevent the concentration of fru-2,6-P_2_ from becoming too high, thereby preventing the metabolic rate from being too fast and maintaining metabolic homeostasis. The mutation of FBPase-2 has also been found to lead to accelerated metabolism of tumor cells (Ros and Schulze, 2013). However, unlike the tumor cells in early research (Saha et al., 2016), in which mitochondrial activity was significantly downregulated, the FBPase-2 mutant in our study (Δ*pfk2-a*) showed more active mitochondrial metabolism than the WT. In addition to the TCA, the expression of AOX was significantly increased in the FBPase-2 mutant (Δ*pfk2-a*). AOX provides an alternative route for electrons passing through the electron transport chain. Although activation of the oxidase reduces ATP generation, it may enhance an organism’s ability to resist stressors via maintenance of the oxidized state of the upstream electron-transport components (Saha et al., 2016). In the FBPase-2 mutant (Δ*pfk2-a*), the increase in mitochondrial metabolic flux also increases the burden on the electron transport chain; as an alternative route, increased expression of AOX contributes to relaxing the tension in the highly coupled electron transport process in mitochondria, thus providing and maintaining mitochondrial metabolic homeostasis.

The evolution of the PFK2/FBPase-2 showed that the bifunctional enzyme seems to have arisen by gene fusion (Rider et al., 2004). Herein, results shown that thus *pfk2s* have both domain in *M. thermophila*, but activities of PFK2 and FBPase-2 are separable, just like *S. cerevisiae*. In *S. cerevisiae*, overexpression of PFK26 (PFK2 activity) significantly down-regulated the intracellular ATP accumulation (Jin et al., 2008; Wu et al., 2022), which could also promote the sugar utilization to a certain extent, and the enhancement of glycolysis also significantly increased the NADH/NAD^+^ ratio (Wu et al., 2022). In our study, overexpression of *pfk2-c* (PFK2 activity) and knockout of the *pfk2-a* (FBPase-2 activity) both promoted glucose metabolism. The reason for the reduced biomass of Δ*pfk2-a* strains in liquid medium is partly due to the production of more byproducts than WT, which reducing the flow of carbon sources to biomass synthesis. On the other hand, the increased expression of mitochondrial AOX in the Δ*pfk2-a* strain was also not conducive to the ATP synthesis and biomass increase. The acceleration of glycolysis rate is conducive to the formation of cytoplasmic NADH (derived from glyceraldehyde 3-phosphate dehydrogenase, GAPDH), and the synthesis of cytoplasmic glycerol and malate requires NADH to provide reducing power. Therefore, the increase in precursor compounds and reducing power promote the increase in the production of glycerol and malate. According to our metabolome results, production of other metabolites in the central carbon metabolism pathway such as succinate and fumarate also increase after knocking out *pfk2-a*.

Compared with prokaryotes and yeast, the filamentous fungi have a slow metabolic rate. Therefore, the strategy of knocking out FBPase-2 activity may be useful to accelerate the metabolic rate and promote the production of metabolites in metabolic engineering of such fungi. Our study showed that this strategy was effective for enhancing the production of multiple metabolites in *M. thermophila*, including glycerol and malate, suggesting that FBPase-2 could be used as a general target for metabolic engineering.

## Data availability statement

The datasets presented in this study can be found in online repositories. The names of the repository/repositories and accession number(s) can be found below: https://www.ncbi.nlm.nih.gov/, GSE214142.

## Author contributions

DH, YZ, and CT conceived the project and wrote the manuscript. DH and YZ performed the metabolic engineering experiments. DH, YZ, DL, and DW analyzed the data. All authors read and approved the final manuscript.

## Supplementary material

Supplementary Figure 1 Predicted domains of PFK2/FBPase-2 in *Myceliophthora thermophila* in the Pfam database (https://pfam.xfam.org/). PFK2 is shown in red; FBPase-2 is shown in blue.

Supplementary Figure 2 Relative amino acid levels in the WT, Δ*pfk2-a*, and Δ*pfk2-abc* strains at days 2, 3, and 4 **(A–C)**. The concentrations of extracted intracellular intermediates were measured by LC–MS/MS and are expressed as normalized peak areas. Values and error bars represent means and standard deviations of independent triplicate experiments, respectively.

Supplementary Figure 3 Phylogenetic tree of the PFK2/FBPase-2 proteins in Ascomycetes. Approximate likelihood-ratio test (Shimodaira–Hasegawa-like) was used for statistical tests. The tree was rooted using the human PFKFB2 (HumPFKFB2) as an out-group. *Myceliophthora thermophila* (Mt), *S. cerevisiae* (Sc), *Chaetomium* sp. (Cha), *Staphylotrichum longicolle* (Stalo), *Madurella mycetomatis* (Mamy), *Podospora comata* (Poco), *Thermochaetoides thermophila* (Thermo), *Neurospora tetrasperma* (Neute), *Diaporthe helianthin* (Diahe), *Coniella lustricola* (Colu).

Supplementary Figure 4 Alignment of the amino acid sequences of *pfk2/fbp2*. Alignments of *pfk2-a*, *pfk2-a*, and *pfk2-c* and three other homologs were conducted in MEGA 7 and GENEDOC. The red asterisk represents the conserved active site RHG motif.
