# Supplementary figures and images for: PFK2/FBPase-2 is a potential target for metabolic engineering in the filamentous fungus Myceliophthora thermophila
Source: Front Microbiol. 2022 Nov 21;13:1056694. doi: 10.3389/fmicb.2022.1056694 (PMC9721465; doi:10.3389/fmicb.2022.1056694)

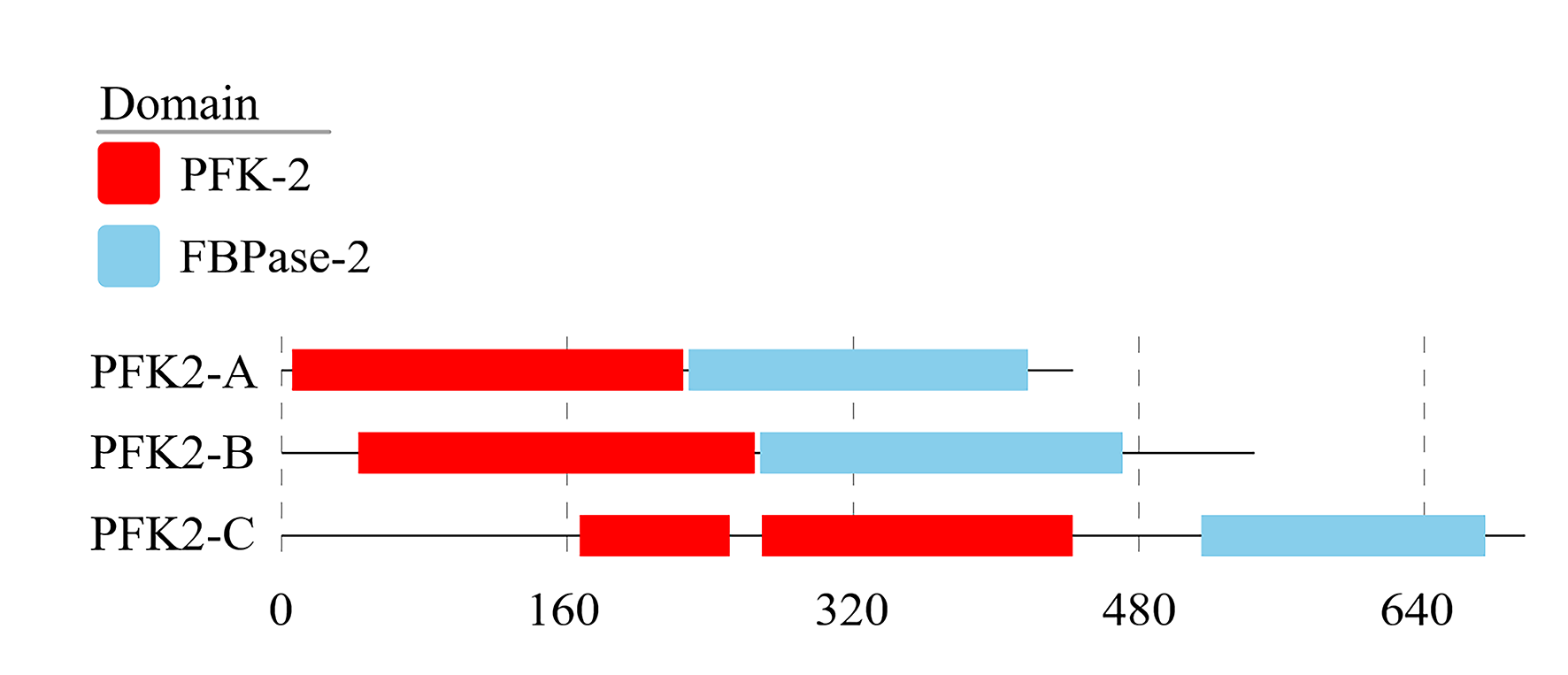

Supplement: Supplementary Figure 1 — Predicted domains of PFK2/FBPase-2 in Myceliophthora thermophila in the Pfam database (https://pfam.xfam.org/). PFK2 is shown in red; FBPase-2 is shown in blue. [file Image_1.TIFF]

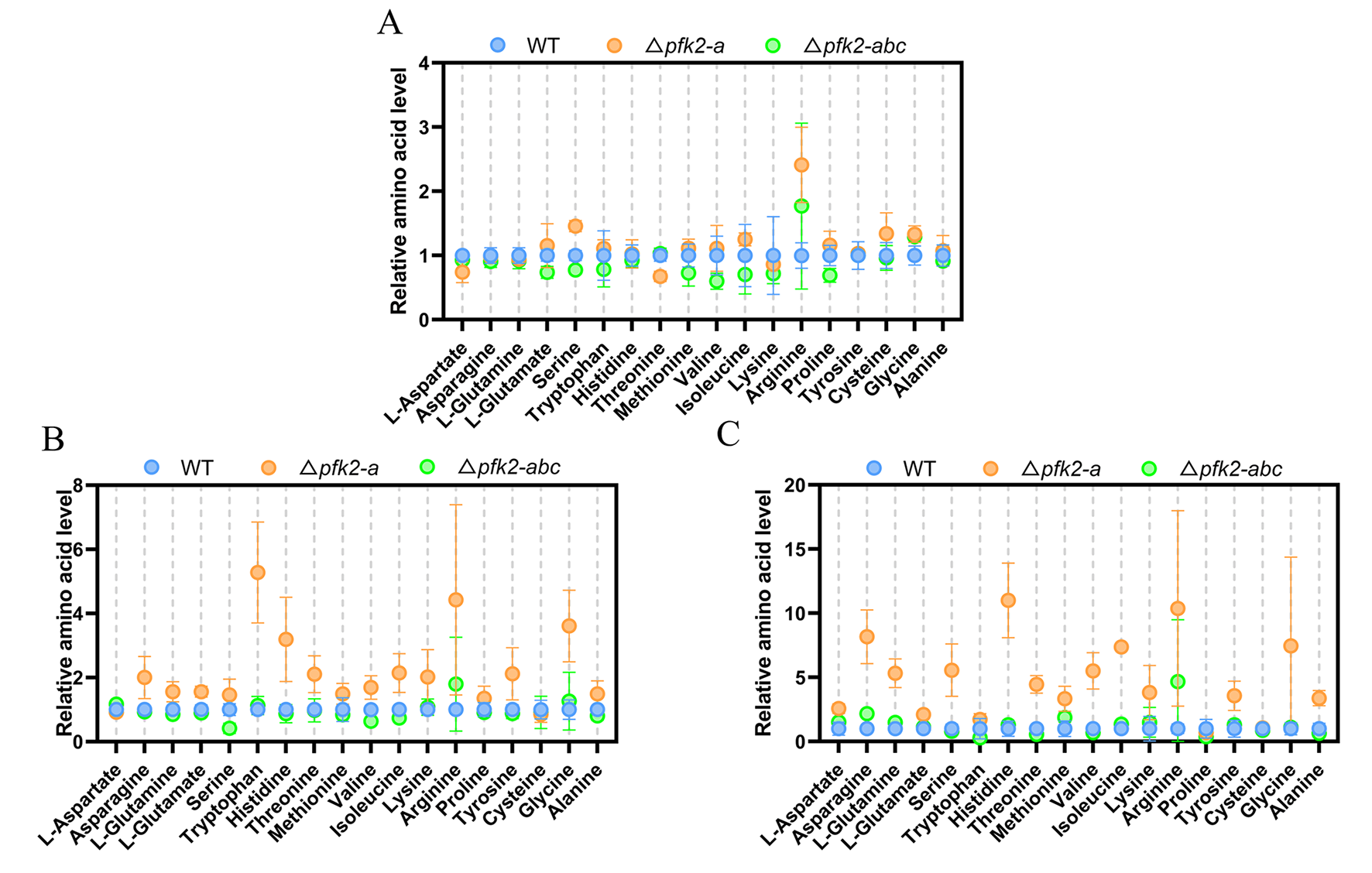

Supplement: Supplementary Figure 2 — Relative amino acid levels in the WT, Δpfk2-a, and Δpfk2-abc strains at days 2, 3, and 4 (A–C). The concentrations of extracted intracellular intermediates were measured by LC–MS/MS and are expressed as normalized peak areas. Values and error bars represent means and standard deviations of independent triplicate experiments, respectively. [file Image_2.TIF]

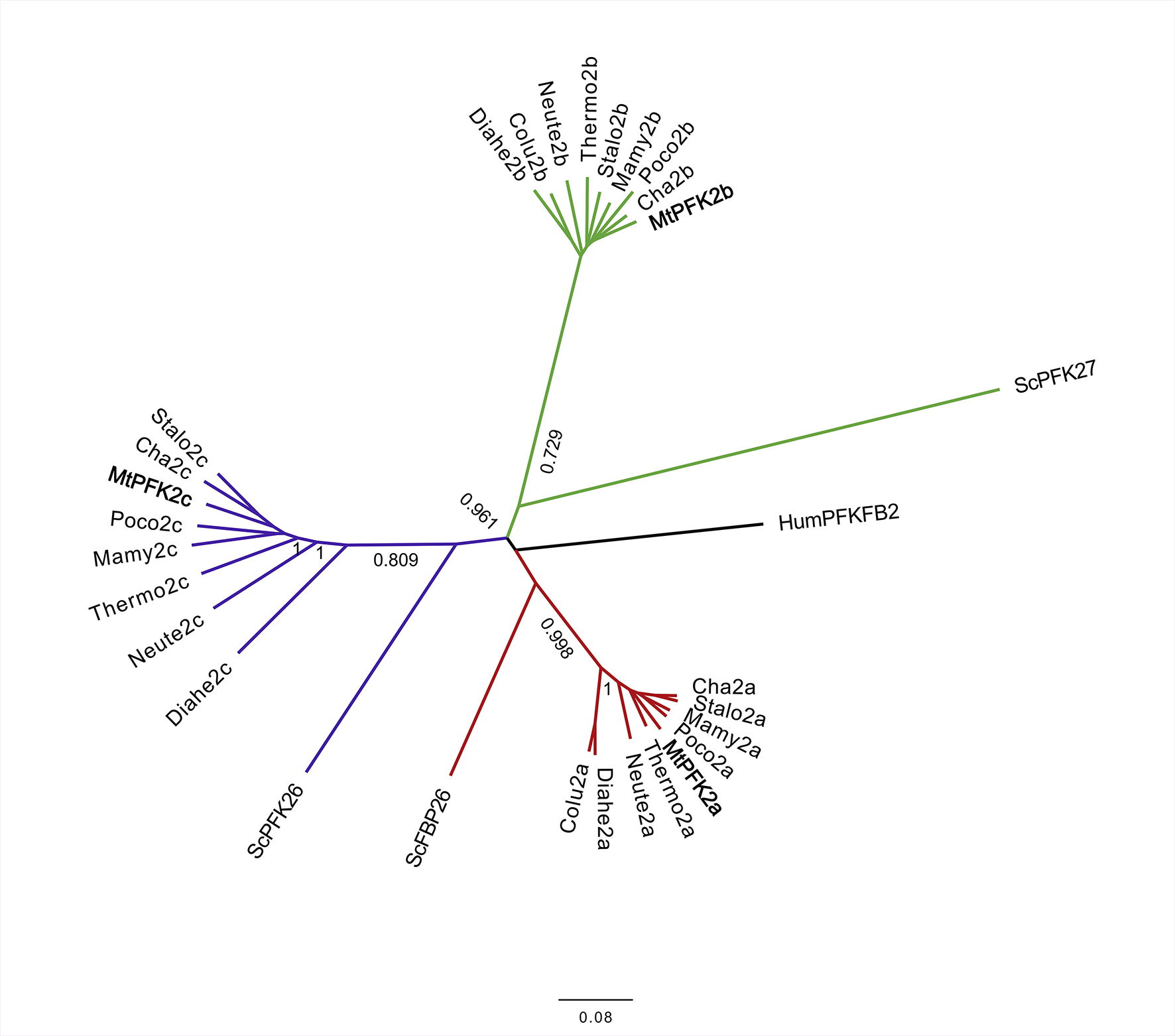

Supplement: Supplementary Figure 3 — Phylogenetic tree of the PFK2/FBPase-2 proteins in Ascomycetes. Approximate likelihood-ratio test (Shimodaira–Hasegawa-like) was used for statistical tests. The tree was rooted using the human PFKFB2 (HumPFKFB2) as an out-group. Myceliophthora thermophila (Mt), S. cerevisiae (Sc), Chaetomium sp. (Cha), Staphylotrichum longicolle (Stalo), Madurella mycetomatis (Mamy), Podospora comata (Poco), Thermochaetoides thermophila (Thermo), Neurospora tetrasperma (Neute), Diaporthe helianthin (Diahe), Coniella lustricola (Colu). [file Image_3.TIF]

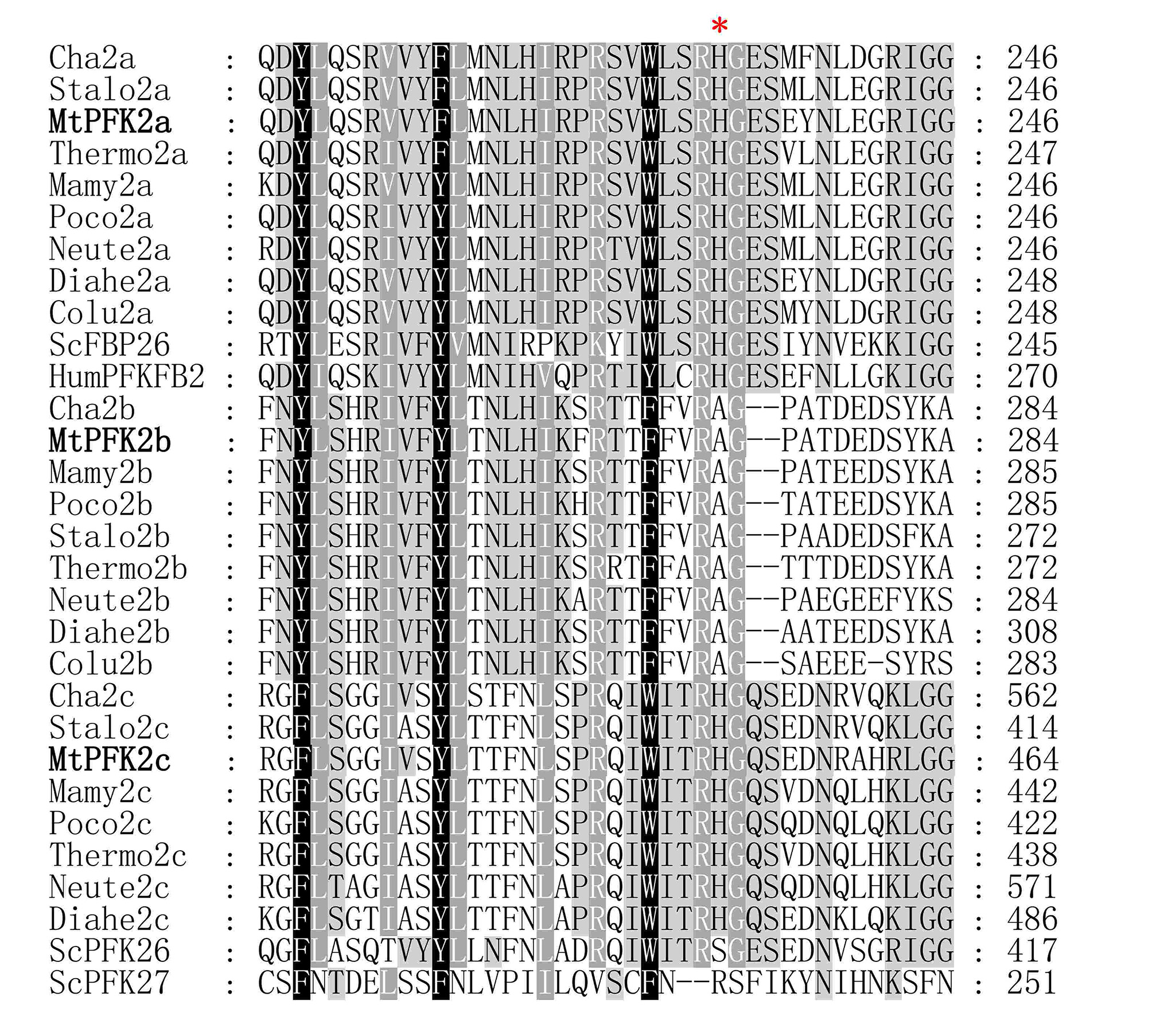

Supplement: Supplementary Figure 4 — Alignment of the amino acid sequences of pfk2/fbp2. Alignments of pfk2-a, pfk2-a, and pfk2-c and three other homologs were conducted in MEGA 7 and GENEDOC. The red asterisk represents the conserved active site RHG motif. [file Image_4.JPEG]
